# Homotypic Targeting of [^89^Zr]Zr-Oxine Labeled PC3 and 4T1 Cells in Tumor-Bearing Mice

**DOI:** 10.3390/pharmaceutics17101259

**Published:** 2025-09-26

**Authors:** Volkan Tekin, Noel E. Archer, Solana R. Fernandez, Hailey A. Houson, Jennifer L. Bartels, Suzanne E. Lapi

**Affiliations:** Department of Radiology, University of Alabama at Birmingham, Birmingham, AL 35294, USA; vtekin@uabmc.edu (V.T.); narcher@uabmc.edu (N.E.A.); sfernandez@uabmc.edu (S.R.F.); hhouson@uabmc.edu (H.A.H.); jburkemper@uabmc.edu (J.L.B.)

**Keywords:** homotypic targeting, cell labeling, tumor-self targeting, cell tracking, oxine, PC3, 4T1

## Abstract

**Background/Objectives**: Homotypic targeting refers to the ability of cells to preferentially interact with other cells of the same type. An understanding of how cells use homotypic targeting (self-homing) characteristics for tumor-targeting purposes may aid in the effective delivery of radionuclides or other drugs for imaging or therapeutic applications. Additionally, studies investigating the targeting properties of cells from the same lineage may shed light on this interesting mechanism, allowing it to be harnessed for other applications. The objective of this study was to assess the tumor-self targeting potential of PC3 prostate cancer and 4T1 breast cancer cells using a direct cell labeling technique, with a focus on evaluation of cellular labeling efficiency, cell viability, cellular efflux, and in vivo tumor-self targeting capability using both identical and dissimilar tumor models. **Methods**: [^89^Zr]Zr-oxine was prepared and utilized for the labeling of PC3 and 4T1 cells. Following the assessment of cell labeling efficacy, viability, and efflux, PET/CT imaging and biodistribution studies were conducted with [^89^Zr]Zr-oxine labeled PC3 and 4T1 cells in PC3 and 4T1 tumor-bearing mice models. **Results**: Both PC3 cells and 4T1 cells were radiolabeled with [^89^Zr]oxine, with PC3 cells illustrating a higher labeling efficiency (86.55 ± 0.38%) than 4T1 cells (46.95 ± 1.47%). Notably, radiolabeled PC3 cells illustrated significant uptake in PC3 tumors (7.54 ± 1.07%ID/gram at 24 h and 6.95 ± 3.56%ID/gram at 48 h) with lower tumor uptake in the 4T1 xenograft model (1.79 ± 0.29%ID/gram at 24 h and 1.42 ± 0.71%ID/gram at 48 h), illustrating the potential of self-targeting. **Conclusions**: Both PC3 and 4T1 cells followed a similar pattern of biodistribution, with labeled PC3 cells demonstrating lower blood retention and reduced uptake in non-target organs such as lungs and heart. Taken together, these results may indicate that PC3 cells illustrate homotypic targeting, warranting further investigation of this phenomenon.

## 1. Introduction

Recently, there has been an enhanced interest in the pursuit of novel tumor-targeting pathways by using labeled cells or constructs coated with cell membranes, which have been shown to have homotypic targeting or self-homing characteristics [1,2,3,4,5]. It is hypothesized that this is due to the fact that tumor cells tend to agglomerate to constitute solid tumors owing to the presence of specific proteins on the surface (focal adhesion proteins, integrin, focal adhesion kinase, and RHO family proteins) [6]. As cancer cells are robust and easy to culture in large volumes, we aimed to investigate this mechanism of selectively targeting cells of the same type, commonly referred to as homotypic targeting, which sets them apart from most other membrane donors [6]. Cell tracking using PET imaging has the potential to elucidate important insights into this phenomenon, where the homotypic tumor cells can be harnessed to achieve cell recognition of cancer cells of the same line [7].

Cell labeling can primarily be categorized into two types: direct cell labeling and indirect cell labeling. The method we selected for our study, direct cell labeling, involves radiolabeling cells prior to their administration. This approach is the most straightforward, enabling the labeling of any cell type without requiring genetic modification or cell surface modification [7]. Alternatively, some studies have utilized DFO-NCS for cell surface attachment. Although this method is effective, changes to the cell surface could impact homing, and thus the direct approach was investigated [7].

8-Hydroxyquinoline (oxine) is known to bind a diverse range of metal ions, which facilitates the formation of neutral, lipophilic metal complexes [8]. Several research groups have used oxine-radiometal complexes such as [^111^In]In-oxine [9], [^68^Ga]Ga-oxine [10], [^64^Cu]Cu-oxine [11], and [^52^Mn]Mn-oxine [12] in both clinical and pre-clinical studies with a focus on cell tracking. The radionuclide ^89^Zr, as [^89^Zr]Zr(oxinate)_4,_ can be used to track cells using Positron Emission Tomography (PET) over an extended time due to its long half-life of 3.27 days [13,14,15,16,17,18].

In the literature, two molecules, cadherins and integrins, represent two of the most extensively researched categories of adhesion receptors. Cadherins facilitate homotypic cell–cell adhesion, while integrins are responsible for the adhesion between a cell and its extracellular matrix [19]. In healthy cells, cadherins and integrins play a crucial role in preserving tissue integrity while offering structural support and essential signals for cellular survival and functionality. Conversely, in cancerous cells, these molecules facilitate invasion, enhance pro-invasive signaling, and contribute to angiogenesis [20]. Although research indicates that these molecules may play a role in homotypic targeting, the precise mechanism remains unclear.

PC3 (human prostate adenocarcinoma cells) and 4T1 (murine mammary carcinoma cells) cell lines were selected with the purpose of investigating potential differences in tumor-targeting of cancer cells derived from different origins (human and murine), tissue (prostate and breast), and sex. The primary biological and molecular characteristics of 4T1 cells that are pertinent to homotypic targeting include their hybrid epithelial/mesenchymal phenotype [21] and elevated levels of specific cell-surface markers such as the epithelial cell adhesion/activating molecule (EpCAM) [22]. The essential biological and molecular traits of PC3 cells pertinent to homotypic targeting encompass their mesenchymal phenotype, the profile of cadherin expression, distinct patterns of integrin expression, their behavior in collective cell migration [23,24] as well as their high expression of the L1 cell adhesion molecule (L1CAM), which directly contributes to their ability to cluster together (homotypic adhesion) and supports their highly metastatic behavior [25].

To evaluate the tumor-self targeting potential of PC3 and 4T1 cancer cells, we used a [^89^Zr]Zr-oxine direct cell labeling technique and evaluated cellular labeling efficiency, cell viability, and cellular efflux. Additionally, the tumor-self targeting capabilities of [^89^Zr]Zr-oxine labeled PC3 and 4T1 cells were assessed using matched PC3 and 4T1 tumor models. Additionally, as PC3 cells illustrated enhanced homotypic targeting, studies were conducted with labeled PC3 cells to evaluate the tumor self-targeting capabilities in PC3 and 4T1 tumor models.

## 2. Materials and Methods

All the reagents were purchased from Thermo Fisher Scientific (Waltham, MA, USA) unless otherwise specified.

### 2.1. Cell Culture

PC3 human prostate adenocarcinoma cells (ATCC, Manassas, VA, USA) were cultured in Dulbecco’s Modified Eagle Medium supplemented with 10% FBS and 0.1% gentamicin. 4T1 murine mammary carcinoma cells (ATCC, Manassas, VA, USA) were cultured in RPMI-1640 Medium supplemented with 10% FBS and 0.1% gentamicin. Cells were incubated at 37 °C and 5% CO_2_.

### 2.2. Preparation of [^89^Zr]Zr-Oxine

Oxine solution was prepared according to Massicano et al. [4]. 5 mg of 8-hydroxyquinoline was dissolved in aqueous solution (7 mL) and dissolved at 80 °C for 10 min. In total, 2.38 g of HEPES was added to the 8-hydroxyquinoline aqueous solution and mixed until completely dissolved. Then, 1 mL of polysorbate (Tween-80) aqueous solution (10 mg/mL) and 0.5 mL of 10 M NaOH were added to the mixture, and the final pH was measured between 7.5 and 8. [^89^Zr]Oxalate was produced at the UAB Cyclotron Facility via ^89^Y(p,n)^89^Zr reaction as previously reported [26]. [^89^Zr]Oxalate was neutralized with 2 M hydrochloric acid (HCl) or 2 M sodium hydroxide (NaOH). 50 µCi (1.85 MBq) of neutralized [^89^Zr]Oxalate solution was added to 0.1 mL of oxine solution and mixed for 30 min (300 rpm) at 37 °C. The quality control of [^89^Zr]Zr-oxine was performed with iTLC using a 50 mM DTPA solution as the mobile phase.

### 2.3. [^89^Zr]Zr-Oxine Labeling of PC3 and 4T1 Cells

5 × 10^5^ of PC3 or 4T1 cells were suspended in 0.5 mL of PBS. 50 µCi (1.85 MBq) of [^89^Zr]Zr-oxine was added to the cell suspension. The cell number was based on literature studies [27]. Cells were incubated at 37 °C for an hour (300 rpm). Cells were centrifuged at 150× *g* for 5 min and rinsed 3 times with warm PBS. After the last rinse, the cell pellet was re-suspended in 0.1 mL of PBS, and the amount of radioactivity was measured with a dose calibrator (Capintec, CRC-55tR, Cal# 505, Mirion, Technologies Inc., Atlanta, GA, USA). Cell viability was evaluated with trypan blue dye; a 20 µL aliquot of the mixture of trypan blue and cell suspension (1:1, *v*:*v*) was loaded into a disposable cell counting chamber slide, and viable cells were counted using a Cellometer Auto T4 Bright Field Cell Counter (Nexcelom, Lawrence, MA, USA).

### 2.4. Cellular Efflux of [^89^Zr]Zr-Oxine Labeled PC3 and 4T1 Cells

[^89^Zr]Zr-oxine labeled PC3 or 4T1 cells were added into 0.1 mL of human blood plasma and incubated for 1, 2, 3, 8, and 24 h at 37 °C (300 rpm). Each aliquot was centrifuged at 150× *g* for 10 min. The supernatants were measured with a HIDEX gamma counter (Lablogic, Clair-Mel City, FL, USA), and the efflux was calculated as follows:

$$Cell\;efflux=\left[\frac{Radioactivity\;in\;supernatant}{Radioactivity\;in\;cell\;pellet+Radioactivity\;in\;supernatant}\right]\times \;100$$

### 2.5. In Vivo Experiments

All animal studies were conducted in compliance with the guidelines for the care and use of research animals established by the University of Alabama at Birmingham’s Institutional Animal Care and Use Committee (IACUC) and approved with the animal protocol number IACUC-22546. Mice were allowed to acclimate for at least a week before any procedures were performed. Mice were kept in cages that included suitable bedding and enrichment; additionally, their water and diet were managed to comply with IACUC criteria. Mice were monitored twice a week to determine if there was any body weight reduction exceeding 30%, if the tumor volume reached 2000 mm^3^, or if any observable dysfunctions occurred.

Male and female athymic nude mice (Charles River, Wilmington, MA, USA, *n* = 4 for each group [27]) were implanted with cells to form PC3 tumors and 4T1 tumors, respectively. The strain of athymic nude mice was also used for the 4T1 tumor model to allow for direct comparisons to the PC3 tumor model. For the tumor implantation, 1 × 10^6^ of PC3 cells in PBS (0.1 mL/per mouse) were subcutaneously injected (either right or left shoulder) to 3–4 weeks old mice 3–4 weeks before the studies and 5 × 10^5^ of 4T1 cells in PBS (0.1 mL/per mouse) were subcutaneously injected (either right or left shoulder) to 6–8 weeks old mice 2 weeks before the studies. The PET/CT imaging was performed when the tumor size reached approximately 150–200 mm^3^.

At each imaging time point, mice were anesthetized with 2–3% isoflurane in oxygen, and approximately 50 µCi (1.85 MBq) of [^89^Zr]Zr-oxine labeled 5 × 10^5^ of PC3 or 4T1 cells (in 0.1 mL/per mouse) were intravenously administered via either lateral tail vein or retro-orbitally (the route was kept consistent within cohorts). [^89^Zr]Zr-oxine labeled PC3 or 4T1 cells were administered within 30 min following the completion of the radiolabeling process. Mice were imaged on a GNEXT small animal PET/CT (Sophie, Springfield, VA, USA) with static PET acquisition (20–30 min depending on the time point) followed by a 3 min CT (80 kVp) at time points up to 7 days. At the end of the imaging, mice were euthanized, and organs were harvested for biodistribution. Organs were weighed and measured using a HIDEX gamma counter. Biodistribution was processed using GraphPad Prism 10. Images were processed and SUVs were calculated using VivoQuant software, 2022, (Invicro, Boston, MA, USA).

### 2.6. Statistics

The analysis of the paired *t*-test was performed on SUV mean data using GraphPad Prism 10, and *p*-values were calculated. Statistical significance is indicated as ns = *p* > 0.05, * = *p* ≤ 0.05, ** = *p* ≤ 0.01, *** = *p* ≤ 0.001, **** = *p* ≤ 0.0001.

## 3. Results

[^89^Zr]Zr-oxine was prepared as previously reported in radiochemical yields of >99% as assessed with iTLC with 50 mM DTPA solution (Supplementary Figure S1) [4]. Labeling yields of [^89^Zr]Zr-oxine-PC3 and [^89^Zr]Zr-oxine-4T1 were 86.55 ± 0.38% (*n* = 10) and 46.95 ± 1.47% (*n* = 10), respectively, and cell viability studies using trypan blue dye demonstrated viability percentages of 96% and 95% for [^89^Zr]Zr-oxine-PC3 and [^89^Zr]Zr-oxine-4T1, respectively. (Supplementary Table S1).

**Figure 1:**
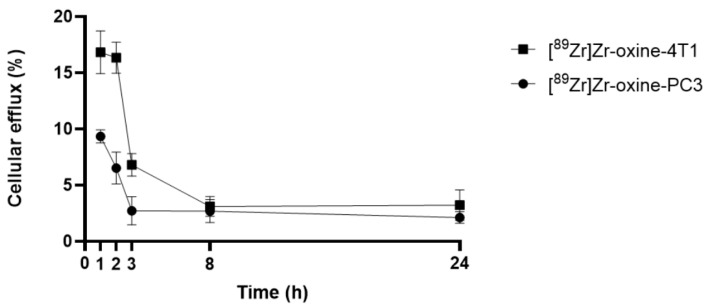
Cellular efflux (%) graph of [^89^Zr]Zr-oxine from PC3 cells and [^89^Zr]Zr-oxine from 4T1 cells.

Cell efflux (%) of [^89^Zr]Zr-oxine from PC3 cells in human blood plasma was 9.34 ± 0.58, 6.53 ± 1.42, 2.72 ± 1.25, 2.69 ± 1.01, and 2.42 ± 0.52% at 1, 2, 3, 8, and 24 h, respectively, as shown in Figure 1. Cell efflux of [^89^Zr]Zr-oxine from 4T1 cells in human blood plasma was 16.83 ± 1.90, 16.34 ± 1.38, 6.81 ± 1.01, 3.11 ± 0.88, and 3.85 ± 1.45% at 1, 2, 3, 8, and 24 h, respectively. The estimated specific activity for injections was 86 µCi/1 × 10^5^ cells for [^89^Zr]Zr-oxine labeled PC3 and 46 µCi/1 × 10^5^ cells for [^89^Zr]Zr-oxine labeled 4T1. This estimate is conservative due to the loss of cells that occurs during the washing step.

To investigate homotypic targeting, [^89^Zr]Zr-oxine-PC3 cells were imaged in PC3 tumor-bearing mice, and [^89^Zr]Zr-oxine-4T1 cells were imaged in 4T1 tumor-bearing mice with 4 h, 24 h, 4 d, and 7 d PET/CT imaging followed by 7 d post-injection biodistribution. An additional study was performed with [^89^Zr]Zr-oxine-PC3 in both PC3 and 4T1 tumor-bearing mice, along with biodistribution studies at 24 and 48 h. The 7 d post injection uptake values of [^89^Zr]Zr-oxine-PC3 in PC3 tumors and [^89^Zr]Zr-oxine-4T1 in 4T1 tumors were 2.30 ± 0.75%ID/g and 1.79 ± 0.23%ID/g, respectively. Furthermore, in biodistribution data, the accumulation of [^89^Zr]Zr-oxine-PC3 in the PC3 tumor model was found to be 7.54 ± 1.07%ID/g at 24 h and 6.95 ± 3.56%ID/g at 48 h, while in the 4T1 tumor model, lower values of 1.79 ± 0.29%ID/g at 24 h and 1.42 ± 0.71%ID/g at 48 h were observed.

**Figure 2:**
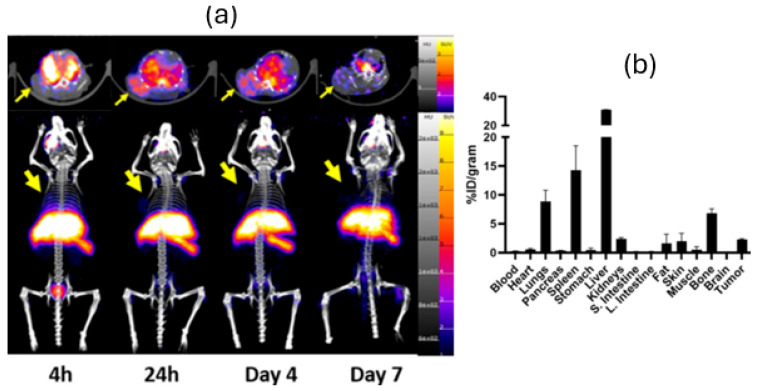
PET/CT images (MIP and transversal) (**a**) and 7-day post-injection biodistribution (**b**) of [^89^Zr]Zr-oxine-PC3 in PC3 tumor-bearing mice. The arrow indicates the location of the tumor.

**Figure 3:**
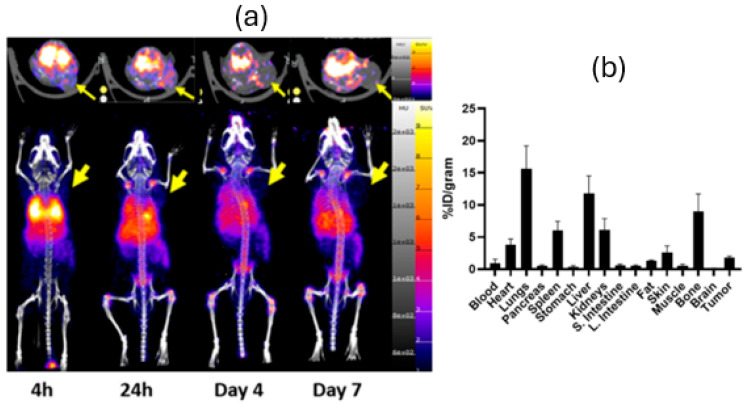
PET/CT images (MIP and transversal) (**a**) and 7-day post-injection biodistribution (**b**) of [^89^Zr]Zr-oxine-4T1 in 4T1 tumor-bearing mice. The arrow indicates the location of the tumor.

PET/CT images and 7 d post-injection biodistribution data (Figure 2) of [^89^Zr]Zr-oxine-PC3 in PC3 tumor-bearing mice illustrated uptake in liver (31.25 ± 2.25%ID/g), spleen (14.65 ± 5.06%ID/g), lungs (8.75 ± 4.12%ID/g), and bone (7.22 ± 1.05%ID/g). PET/CT images and 7 d post-injection biodistribution data (Figure 3) of [^89^Zr]Zr-oxine-4T1 in 4T1 tumor-bearing mice illustrated similar uptake in lungs (15.63 ± 3.56%ID/g), liver (11.82 ± 2.70%ID/g), bone (9.01 ± 2.70%ID/g), and kidneys (6.13 ± 1.73%ID/g).

**Figure 4:**
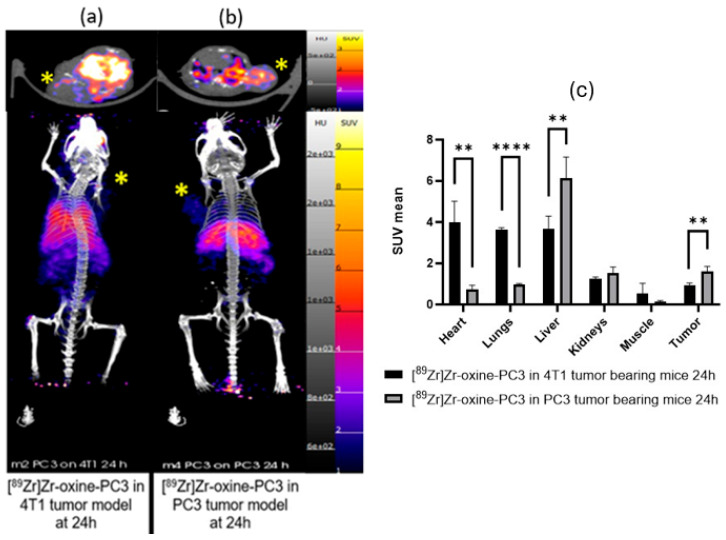
PET/CT images (MIP and transversal) of [^89^Zr]Zr-oxine-PC3 in 4T1 tumor (**a**), PC3 tumor-bearing mice (**b**), and SUV mean comparison at 24 h (**c**). * Tumor. ** = *p* ≤ 0.01, **** = *p* ≤ 0.0001.

**Figure 5:**
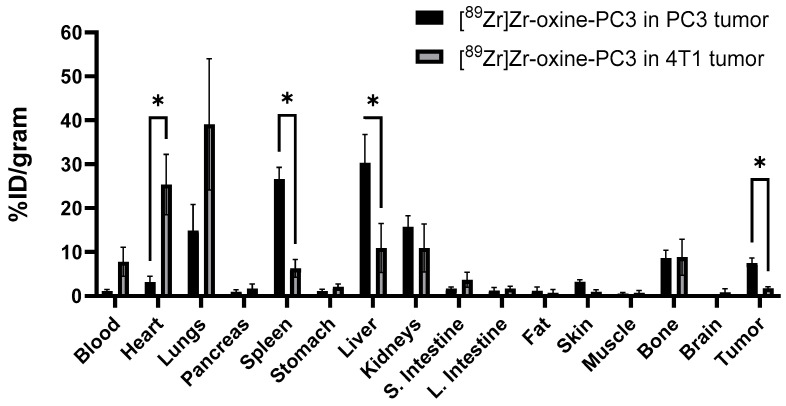
24 h post-injection biodistribution comparison of [^89^Zr]Zr-oxine-PC3 in PC3 tumor and 4T1 tumor-bearing mice. GraphPad Prism 10.3.1, * indicates statistically different differences via multiple unpaired *t* test, heart *p* = 0.0006, spleen *p* = 0.00001, liver *p* = 0.0039, tumor *p* = 0.0001.

PET/CT images and SUV mean values of [^89^Zr]Zr-oxine-PC3 in 4T1 tumor-bearing mice and PC3 tumor-bearing mice at 24 and 48 h are shown in Figure 4 and Supplemental Figure S4, respectively. Tumor accumulation of labeled PC3 cells in the PC3 tumor model was approximately 4-fold higher at 24 h (7.45 ± 1.94 vs. 1.79 ± 1.25%ID/g) and 4.5-fold higher at 48 h (6.95 ± 1.91 vs. 1.42 ± 1.03%ID/g) in comparison to uptake in 4T1 tumors. The biodistribution data for [^89^Zr]Zr-oxine-PC3 (Figure 5, Supplemental Figure S5, and Supplemental Table S3) indicate uptake in the lung (39.05 ± 12.03 and 27.65 ± 7.65%ID/g at 24 and 48 h, respectively) and heart (25.39 ± 5.92 and 27.65 ± 7.65% ID/g at 24 and 48 h, respectively). The lung uptake for [^89^Zr]Zr-oxine-PC3 in the PC3 tumor model was 14.86 ± 5.14%ID/g at 24 h and 15.24 ± 3.90%ID/g at 48 h. The heart uptake for [^89^Zr]Zr-oxine-PC3 in the PC3 tumor model was 3.15 ± 1.13%ID/g at 24 h and 3.23 ± 3.21%ID/g at 48 h. The uptake [^89^Zr]Zr-oxine-PC3 in spleen (26.65 ± 2.28%ID/g at 24 h and 28.65 ± 12.40%ID/g at 48 h) and liver (30.31 ± 5.60%ID/g at 24 h and 44.85 ± 9.91%ID/g at 48 h) was observed to be higher in the PC3 model than in the 4T1. An increase in bone uptake was observed for both PC3 and 4T1 tumor models at 48 h (14.07 ± 2.04 and 4.97 ± 1.01%ID/g for PC3 and 4T1 model, respectively).The tumor to blood ratios for [^89^Zr]Zr-oxine-PC3 in the PC3 tumor model were 6.66 (24 h) and 2.24 (48 h). The tumor to blood ratios for [^89^Zr]Zr-oxine-PC3 in the 4T1 tumor model were 0.23 (24 h) and 0.21 (48 h). For [^89^Zr]Zr-oxine-PC3, higher accumulation was noted in PC3 tumors as compared to 4T1, alongside a reduced uptake in the lungs and bones in the PC3 tumor model. The significant lung uptake and blood retention observed in the 4T1 cells point to the potential for micrometastasis, and additional studies with various cell lines should be conducted to investigate this limitation.

PET/CT images and 7 d post-injection of [^89^Zr]Zr-oxine-PC3 in non-tumor mice are shown in Supplemental Figure S2. The distribution and elimination of [^89^Zr]Zr-oxine-PC3 cells exhibited a similar pattern to the tumor-bearing mice, characterized by signal in the lungs at the early time point of 4 h, followed by a subsequent clearance through the liver.

## 4. Discussion

[^89^Zr]Zr-oxine was prepared in high RCYs in agreement with previous reports [4,12,13,14,15,16,17]. PC3 and 4T1 cells were readily labeled with [^89^Zr]Zr-oxine in yields of 86.55 ± 0.38% and 46.95 ± 1.47%, respectively. The disparity in cell labeling efficiency between PC3 and 4T1 cells may be attributed to variations in cell size. This observation aligns with recent findings reported by Sato et al., which established a correlation between the labeling efficacy of [^89^Zr]Zr-oxine and cell size, indicating that the largest cells exhibited the highest uptake [14]. Additionally, cell labeling efficacy can be attributed to the diversity of intracellular proteins. While we hypothesize these differences in protein expression or cell size [14] may be the reason for the labeling efficiency differences, further studies are warranted to better understand this effect. Once the oxine-radiometal complex enters the cell, the radiometal may undergo trans chelation by various intracellular proteins and macromolecules, leading to the retention of the radionuclide within the cell [7].

[^89^Zr]Zr-oxine-PC3 exhibited higher label retention in human blood plasma in comparison to [^89^Zr]Zr-oxine-4T1 (the cell efflux at 3 h was 2.72 ± 1.25% for [^89^Zr]Zr-oxine-PC3, and 6.81 ± 1.01% for [^89^Zr]Zr-oxine-4T1). A decrease in cellular efflux % in both cell lines was observed within the initial eight-hour period, followed by retention up to 24 h. This agrees with Friberger et al., where [^89^Zr]Zr-(oxinate)_4_ labeled macrophages showed a decrease in radioactive retention mostly within 2 days and then remained relatively stable up to 4 days. In their study, the researchers reported a decrease of 16  ±  1.8% in radiolabel retention after 24 h, which is similar to values obtained in this study [27].

Prior to exploring advancements in homotypic targeting across various tumors, we initially examined PC3 cells within PC3 tumors and 4T1 cells within 4T1 tumors. The findings indicated that 4T1 cells were less favorable for homotypic targeting compared to PC3 cells. Consequently, we focused solely on PC3 cells to investigate the distinctions between homotypic targeting and tumor targeting across different tumor types. To investigate homotypic targeting, we administered [^89^Zr]Zr-oxine-PC3 to PC3 and 4T1 tumor-bearing mice under identical conditions. This was conducted with the aim of elucidating any disparities in the behavior of cancer cells when specifically targeting homotypic and heterotypic tumors. We observed tumor-self targeting of PC3 cells in the PC3 tumor model with lower uptake in the mismatch scenario (PC3 cells showed lower uptake in the 4T1 model illustrated by significantly different biodistribution of [^89^Zr]Zr-oxine-PC3 cells in PC3 and 4T1 tumor models 24 h post-injection tumor in PC3 model: 7.45 ± 1.94%ID/g, in 4T1 model tumor 1.79 ± 0.29%ID/g (*p* < 0.001, ANOVA multiple unpaired *t* test). 24 h post-injection SUV mean in PC3 model: 1.61 ± 0.23%ID/g in 4T1 model: 0.94 ± 0.10%ID/g (the significance in differences between tumor, heart, liver, lungs were *p* < 0.05, ANOVA paired *t* test). The tumor to blood ratios for [^89^Zr]Zr-oxine-PC3 in the PC3 tumor model (6.66) were higher than the tumor to blood ratios for [^89^Zr]Zr-oxine-PC3 in the 4T1 tumor model (0.23).

PET/CT images of [^89^Zr]Zr-oxine-PC3 in 4T1 and PC3 tumor-bearing mice also showed [^89^Zr]Zr-oxine-PC3 uptake in other organs. The lung uptake of labeled PC3 cells was lower in the PC3 tumor model in comparison to the 4T1 model. PET/CT images and 7-day post-injection biodistribution data indicate that in the PC3 tumor model, [^89^Zr]Zr-oxine-PC3 was partially cleared out through the kidneys within 4 h; however, a significant signal was observed in the liver over 7 days. Additionally, uptake in the lungs and spleen was observed as expected. This finding aligns with a common pattern observed in cells administered in vivo as reported by Friberger et al. [27]. Abou et al. reported on the accumulation of free ^89^Zr in the bones of mice. The bone uptake, especially at late time points, is likely attributed to the release of free ^89^Zr [28]. To minimize the bone uptake due to free ^89^Zr, alternative cell labeling methods could be investigated. Alternatively, Sato et al. [13] infused DFO to chelate and accelerate the urinary elimination of extracellular free ^89^Zr. This could be considered to minimize the signal of free ^89^Zr in future studies. For [^89^Zr]Zr-oxine-4T1, an accumulation was observed in lungs especially at 4 and 24 h time points; also, uptake in liver and spleen was lower compared to [^89^Zr]Zr-oxine-PC3. The difference in accumulation in lungs can be attributed to the diameter of cells (PC3 cells, 16.6 ± 2.9 µm [29] and 4T1 cells, 8.8 ± 1.3 μm [30]), and the pulmonary uptake and deposition depend on the size. The smaller 4T1 cells may be more readily captured by the lungs compared to the larger PC3 cells. However, additional studies would shed light on this mechanism.

Based on the findings of this research, future studies will focus on further cell lines and the mechanisms involved in homotypic targeting. The main impact of this research is to investigate the underlying biology and the mechanism of the homotypic targeting abilities of cancer cells. Given the uptake in liver, spleen, and bone marrow, these techniques may be limited to diseases not involving these sites, and additional studies are required to understand the future clinical implications of tumor self-targeting.

## 5. Conclusions

Significantly, radiolabeled PC3 cells demonstrated considerable uptake in PC3 tumors, whereas the 4T1 xenograft model exhibited lower tumor uptake (%ID/gram), highlighting the potential for self-targeting. Both PC3 and 4T1 cells exhibited a comparable biodistribution pattern, with labeled PC3 cells showing diminished blood retention and lower uptake in non-target organs, including the lungs and heart, alongside an increase in liver uptake. Despite the fact that only two tumor models were evaluated in immunodeficient mice, these findings suggest that PC3 cells may demonstrate homotypic targeting, which merits further exploration of this phenomenon.

## Data Availability

The raw data supporting the conclusions of this article will be made available by the authors on request.

## Supplementary Materials

The following supporting information can be downloaded at: https://www.mdpi.com/article/10.3390/pharmaceutics17101259/s1, Table S1: Cell labeling yield (%) and cell viability ratio (%) of [^89^Zr]Zr-oxine-PC3 and [^89^Zr]Zr-oxine-4T1; Figure S1. TLC chromatograms of [89Zr]Zr-oxine (a) and 89Zr-oxalate (free 89Zr) (b) (Retention factor values; 0.2 for [89Zr]Zr-oxine and 0.9 for 89Zr-oxalate); Figure S2: PET/CT images (MIP) and 7 d post injection % ID/gram graph of [^89^Zr]Zr-oxine-PC3 in non-tumor mice; Figure S3: PET/CT comparison of [^89^Zr]Zr-oxine-PC3 in 4T1 tumor model, [^89^Zr]Zr-oxine-PC3 in PC3 tumor model and [^89^Zr]Zr-oxine-4T1 in 4T1 tumor model; Figure S4: (a) PET/CT images (MIP and transversal) of [^89^Zr]Zr-oxine-PC3 in 4T1 tumor bearing mice, (b) PET/CT images (MIP and transversal) of [^89^Zr]Zr-oxine-PC3 in PC3 tumor bearing mice, (c) SUV mean comparison of [^89^Zr]Zr-oxine-PC3 in PC3 and 4T1 tumor bearing mice at 48 h post injection. * Tumor; Figure S5: 48 h post injection biodistribution comparison of [^89^Zr]Zr-oxine-PC3 in 4T1 tumor and PC3 tumor bearing mice; Table S2: SUV mean and ID/gram values [^89^Zr]Zr-oxine-PC3; Table S3: The comparison of [^89^Zr]Zr-oxine-PC3 ID/gram values in individual biodistribution studies.

## Abbreviations

The following abbreviations are used in this manuscript:

PC3	Human prostate adenocarcinoma cells
4T1	Murine mammary carcinoma cells
TLC	Thin-layer chromatography
HPLC	High-performance liquid chromatography
ATCC	American type culture collection
SUV	Standardized uptake value
PET/CT	Positron Emission Tomography/Computed Tomography
%ID/gram	Injected dose per gram
