# Supplementary material for: Homotypic Targeting of [89Zr]Zr-Oxine Labeled PC3 and 4T1 Cells in Tumor-Bearing Mice
Source: Pharmaceutics. 2025 Sep 26;17(10):1259. doi: 10.3390/pharmaceutics17101259 (PMC12566915; doi:10.3390/pharmaceutics17101259)
Supplement: Supplementary file 1 [file pharmaceutics-17-01259-s001.zip › pharmaceutics-3861912-supplementary.pdf]

pharmaceutics

Article

Homotypic Targeting of [^{89}Zr]Zr-Oxine Labeled PC3 and 4T1 Cells in Tumor-Bearing Mice

Volkan Tekin, Noel E. Archer, Solana R. Fernandez, Hailey A. Houson, Jennifer L. Bartels and Suzanne E. Lapi *

Department of Radiology, University of Alabama at Birmingham, Birmingham, AL 35294, USA; vtekin@uabmc.edu (V.T.); narcher@uabmc.edu (N.E.A.); sfernandez@uabmc.edu (S.R.F.); hhouson@uabmc.edu (H.A.H.); jburkemper@uabmc.edu (J.L.B.)
* Correspondence: lapi@uab.edu

Supplemental Information

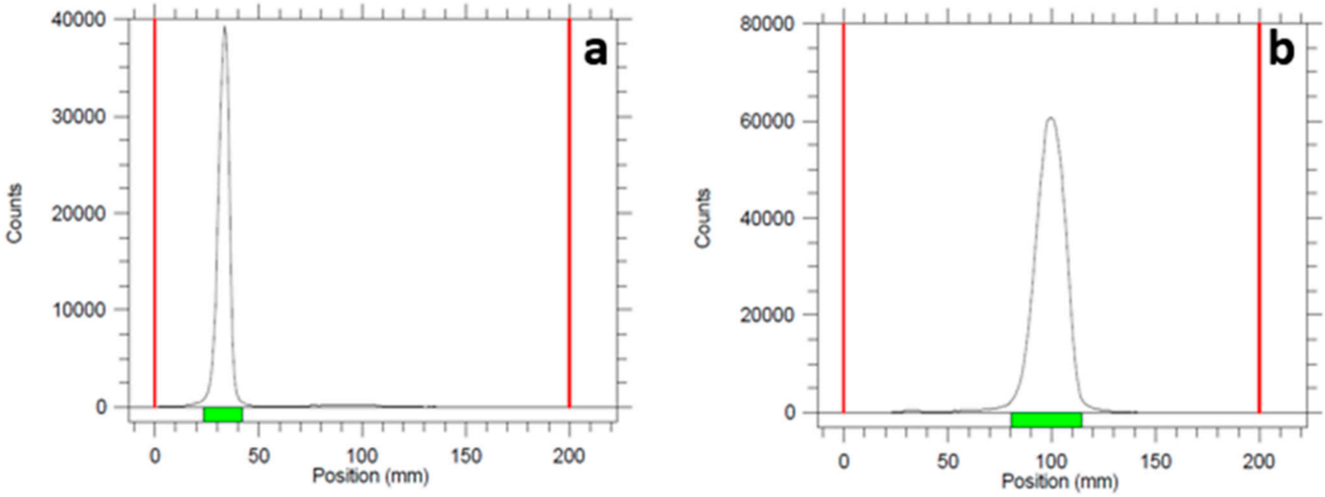

Figure S1. TLC chromatograms of [^{89}Zr]Zr-oxine (a) and ^{89}Zr-oxalate (free ^{89}Zr) (b) (Retention factor values; 0.2 for [^{89}Zr]Zr-oxine and 0.9 for ^{89}Zr-oxalate).

Table S1. Cell labeling yield (%) and cell viability ratio (%) of [^{89}Zr]Zr-oxine-PC3 and [^{89}Zr]Zr-oxine-4T1

	After incubation of labeling		After 1st rinse of PBS		After 2nd rinse of PBS		After 3rd rinse of PBS	
Labeled samples (n=3)	Cell labeling yield (%)	Viability (%)	Cell labeling yield (%)	Viability (%)	Cell labeling yield (%)	Viability (%)	Cell labeling yield (%)	Viability (%)
[^{89}Zr]Zr-oxine-PC3	87.63±0.83	98±1	87.33±0.36	97±1	86.95±0.34	98±2	86.55±0.38	96±1
[^{89}Zr]Zr-oxine-4T1	52.93±2.13	98±1	48.75±1.67	95±1	47.64±1.47	95±1	46.95±1.47	95±1

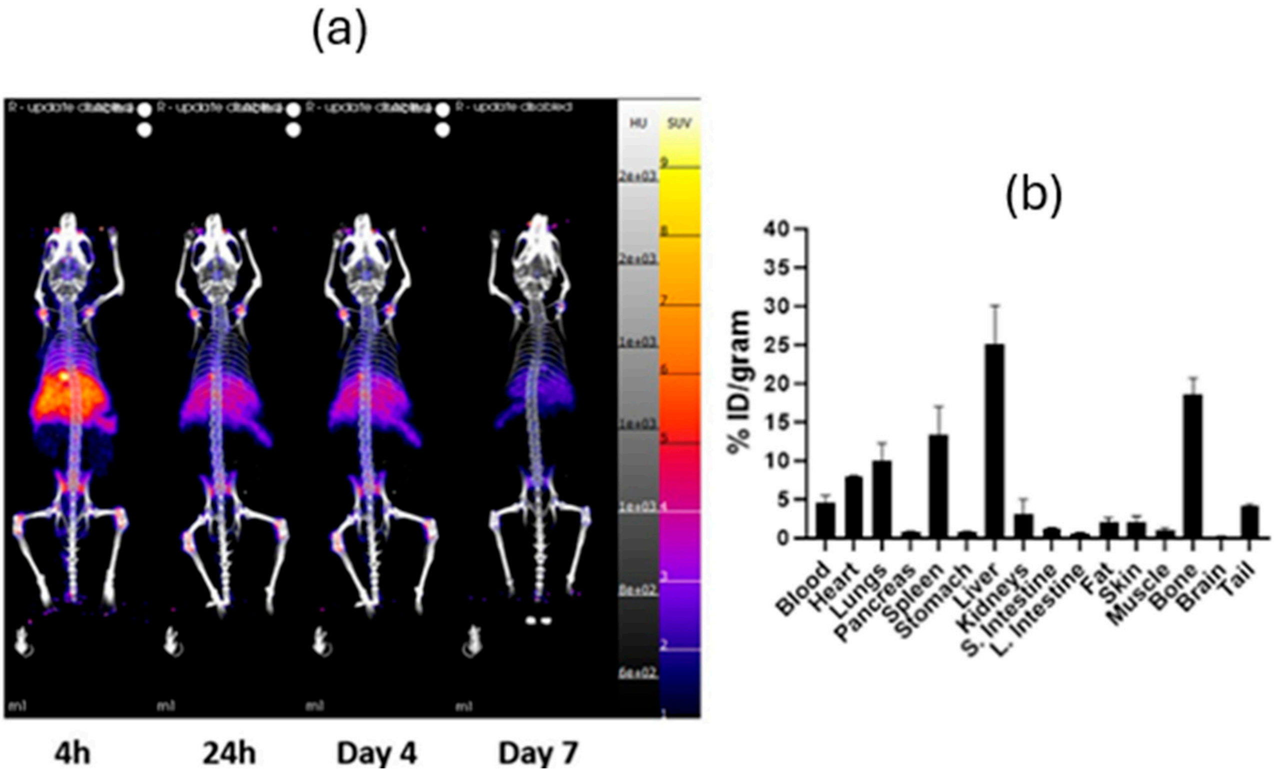

Figure S2. PET/CT images (MIP) (a) and 7d post injection % ID/gram graph (b) of [^{89}Zr]Zr-oxine-PC3 in non-tumor mice.

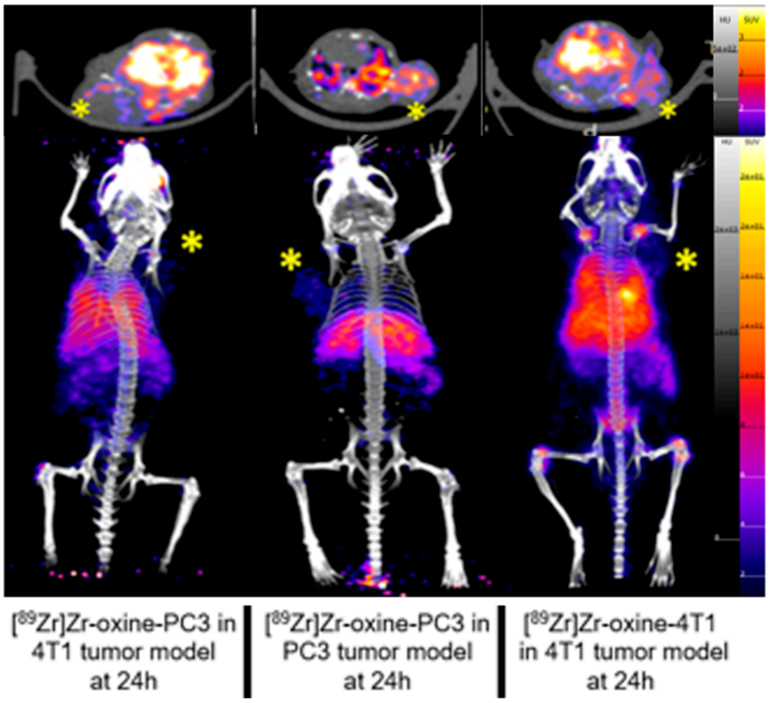

Figure S3. PET/CT comparison of [^{89}Zr]Zr-oxine-PC3 in 4T1 tumor model, [^{89}Zr]Zr-oxine-PC3 in PC3 tumor model and [^{89}Zr]Zr-oxine-4T1 in 4T1 tumor model.

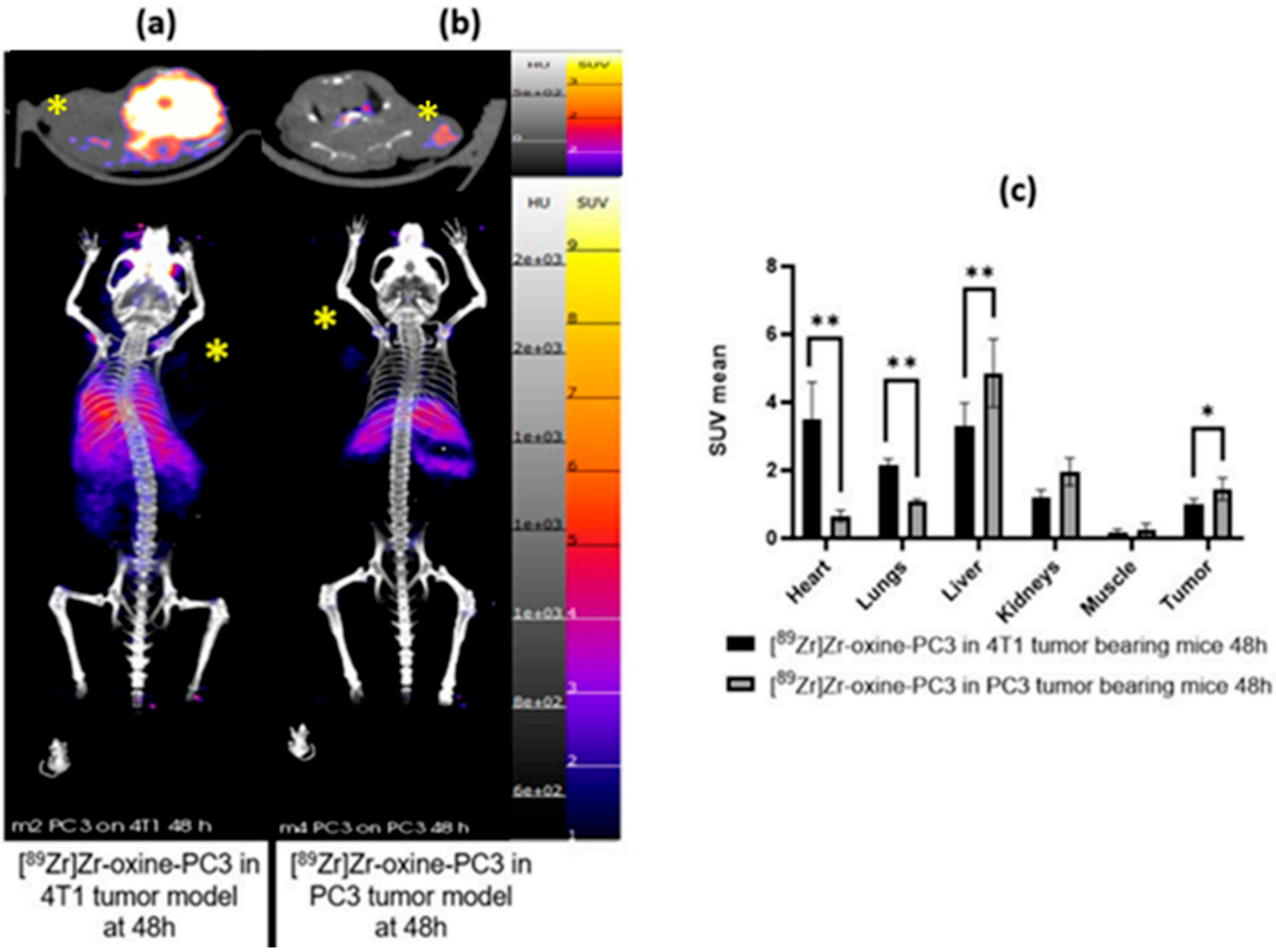

Figure S4. (a) PET/CT images (MIP and transversal) of [^{89}Zr]Zr-oxine-PC3 in 4T1 tumor bearing mice, (b) PET/CT images (MIP and transversal) of [^{89}Zr]Zr-oxine-PC3 in PC3 tumor bearing mice, (c) SUV mean comparison of [^{89}Zr]Zr-oxine-PC3 in PC3 and 4T1 tumor bearing mice at 48h post injection. * Tumor. The significance in difference for tumor, heart, liver, lungs were $p<0.05$, ANOVA paired t test.

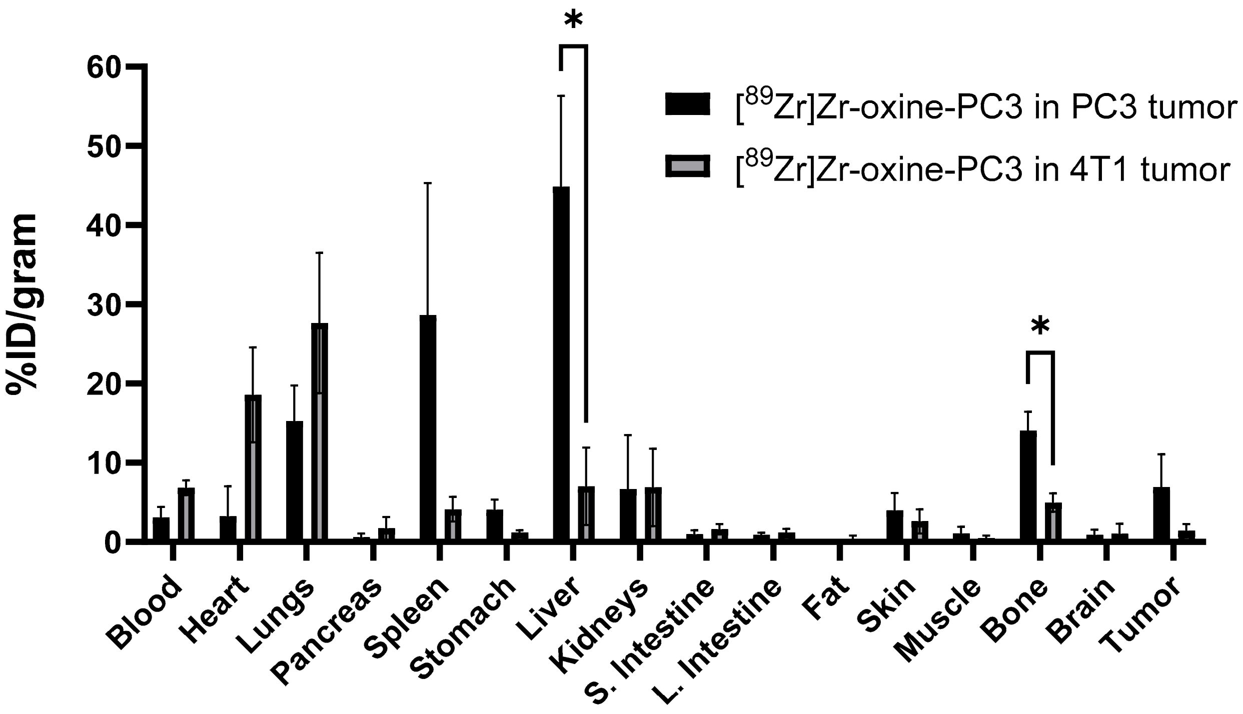

Figure S5. 48h post injection biodistribution comparison of [^{89}Zr]Zr-oxine-PC3 in 4T1 tumor and PC3 tumor bearing mice. GraphPad Prism 10.3.1, multiple unpaired t test, liver p= 0.0009, bone p= 0.0004.

Table S2. SUV mean and ID/gram values [^{89}Zr]Zr-oxine-PC3

[^{89}Zr]Zr-oxine-PC3		PC tumor model		4T1 tumor model	
		SUV mean	ID/gram	SUV mean	ID/gram
24h	Tumor	1.61±0.23	7.54±1.07	0.94±0.10	1.79±0.29
	Lungs	0.96±0.05	14.86±5.14	3.65±0.07	39.05±12.03
	Heart	0.74±0.19	3.15±1.13	3.97±1.04	25.39±5.92
	Liver	6.12±10.3	30.31±5.60	3.69±0.60	10.92±4.85
	Kidneys	1.51±0.30	15.73±2.20	1.25±0.08	10.93±4.71

Table S3. The comparison of [^{89}Zr]Zr-oxine-PC3 ID/gram values in individual biodistribution studies.

		[^{89}Zr]Zr-oxine-PC3		
Timepoint	Organ/ Tissue	PC3 tumor bearing mice	4T1 tumor bearing mice	Non-tumor mice
24h	Tumor	7.54±1.07	1.79±0.29	
	Lungs	14.86±5.14	39.05±12.03	
	Heart	3.15±1.13	25.39±5.92	
	Liver	30.31±5.60	10.92±4.85	
	Kidneys	15.73±2.20	10.93±4.71	
	Bone	8.63±1.57	8.83±3.54	
48h	Tumor	6.95±3.56	1.42±0.71	
	Lungs	15.24±3.90	27.65±7.65	
	Heart	3.23±1.29	18.56±5.18	
	Liver	44.85± 9.91	7.02±4.24	
	Kidneys	6.67±5.92	6.89±4.23	
	Bone	14.07±2.04	4.97±1.01	
7d	Tumor	2.30±0.23		N/A
	Lungs	8.12±2.11		9.99±2.27
	Heart	1.66±0.33		7.96±0.10
	Liver	32.56±1.85		25.02±5.06
	Kidneys	2.65±0.83		3.16±1.84
	Bone	6.96±1.48		18.59±2.11
